# Variable step size methods for solving simultaneous algebraic reconstruction technique (SART)-type CBCT reconstructions

**DOI:** 10.18632/oncotarget.17385

**Published:** 2017-04-24

**Authors:** Heui Chang Lee, Bongyong Song, Jin Sung Kim, James J. Jung, H. Harold Li, Sasa Mutic, Justin C. Park

**Affiliations:** ^1^ Weldon School of Biomedical Engineering, Purdue University, West Lafayette, Indiana, USA; ^2^ J Crayton Pruitt Family Department of Biomedical Engineering, University of Florida, Gainesville, Florida, USA; ^3^ Department of Radiation Medicine and Applied Sciences, University of California San Diego, La Jolla, California, USA; ^4^ Department of Radiation Oncology, Yonsei Cancer Center, Yonsei University College of Medicine, Seoul, Korea; ^5^ Department of Radiation Oncology, University of Florida, Gainesville, Florida, USA; ^6^ Department of Radiation Oncology, Washington University School of Medicine, St. Louis, Missouri, USA

**Keywords:** SART, weighted least squares, image reconstruction, GPU, IGRT

## Abstract

Compared to analytical reconstruction by Feldkamp-Davis-Kress (FDK), simultaneous algebraic reconstruction technique (SART) offers a higher degree of flexibility in input measurements and often produces superior quality images. Due to the iterative nature of the algorithm, however, SART requires intense computations which have prevented its use in clinical practice. In this paper, we developed a fast-converging SART-type algorithm and showed its clinical feasibility in CBCT reconstructions. Inspired by the quasi-orthogonal nature of the x-ray projections in CBCT, we implement a simple yet much faster algorithm by computing Barzilai and Borwein step size at each iteration. We applied this variable step-size (VS)-SART algorithm to numerical and physical phantoms as well as cancer patients for reconstruction. By connecting the SART algebraic problem to the statistical weighted least squares problem, we enhanced the reconstruction speed significantly (i.e., less number of iterations). We further accelerated the reconstruction speed of algorithms by using the parallel computing power of GPU.

## INTRODUCTION

In recent years, the introduction of cone-beam computed tomography (CBCT) in radiation therapy has enabled precise on-line positioning (and on-line/off-line re-planning) of patients [1, 2]. This is possible due to the wealth of information contained in the three-dimensional (3D)-CBCT images including 1) anatomical information [1, 2], 2) geometrical information [3, 4], and 3) CT numbers for possible dose calculations for treatment verifications and plan re-optimizations [5, 6].

Filtered backprojection (i.e., Feldkamp-Davis-Kress algorithm (FDK) for 3D-CBCT [7]) has been the most widely used reconstruction method, but there has been attempts to improve the quality of image with iterative techniques. Simultaneous algebraic reconstruction technique (SART) proposed by Anderson and Kak [8] had a significant impact in the CT imaging field. Compared to ART [9], SART algorithm showed a major advantage especially when samples were incomplete and noisy. Given the non-negative characteristic of imaging coefficients, SART was proved to converge and the sequence generated by SART was represented as a weighted least squares problem [10]. Several variants of SART such as ordered-subset (OS)-SART [11] have been proposed since then mainly to improve the rate of convergence. These studies employed various step-size computation methods and demonstrated the importance of choosing the step-size for enhancing the rate of convergence and computational complexity per iteration [12, 13]. Since SART is essentially a solution to weighted least squares problem, these step step-size computation methods can be directly applied to SART algorithms. When a sufficient number of projections are available, SART with fast step-size computation will be of favorable choice for image reconstruction.

In this work, we propose a novel variable step-size (VS)-SART algorithm that handles the least squares problem based on the Barzilai and Borwein (BB) formulation [14, 15]. First, VS-SART algorithms were formulated as iterative algorithms for solving the objective function. Then, various step-size computation methods including the BB approach were tested with the Shepp-Logan phantom on the image quality as well as the computational complexity. Reconstructed image quality of CatPhan 600 phantom and pancreatic/prostate cancer patients were also evaluated to ensure the consistency of VS-SART algorithms. We envision that our fast-converging algorithm along with advancements in GPU technology will even further reduce the total CT reconstruction time and minimize the computational burden for real-time applications.

Notations. Matrices are denoted by boldface uppercase letters and vectors are denoted by boldface lowercase letters. For a given matrix **A**, the *i*-th column vector is denoted by $a_{i}$, the *j*-th row vector is denoted by $a_{j}^{}$, and the (*i*, *j*)-th element is denoted by $a_{ij}$. For a given vector x, the *n*-th element is denoted by $x_{n}$. Superscript ${\left(\cdot \right)}^{T}$ is used to denote the transpose of a matrix or a vector.

## RESULTS

**Figure 1:**
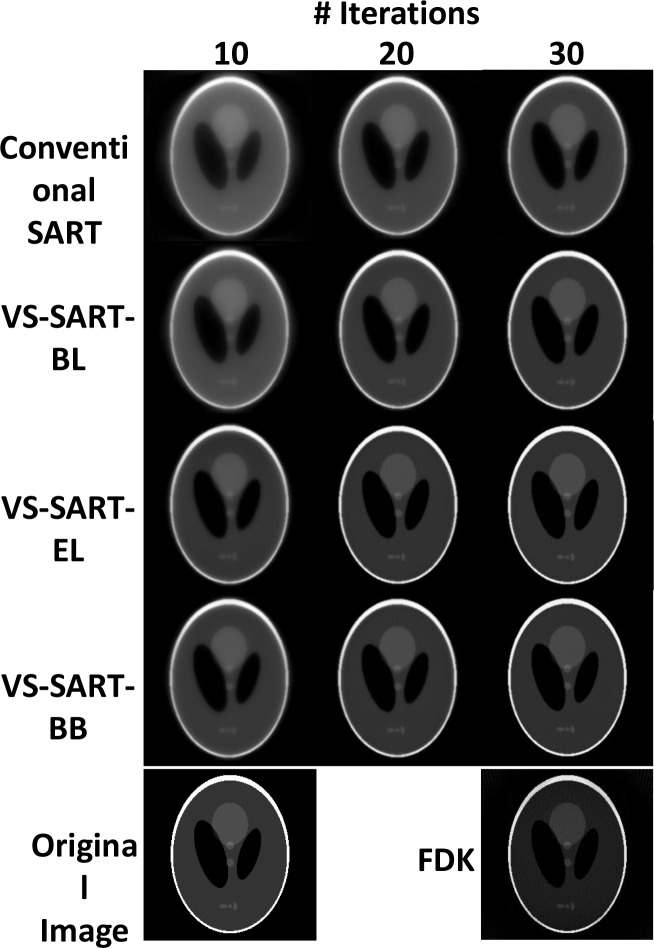
Reconstructed Shepp-Logan phantom images using Conventional SART (α = 1.2), VS-SART-BL, VS-SART-EL, and VS-SART-BB with 10, 20, and 30 iterations These images are compared with the original image and a FDK reconstructed image. A total of 180 projections from 360-degree angle (fan-beam geometry) was used for reconstructions.

Figure 1 illustrates the image quality of reconstructed Shepp-Logan phantom using Conventional SART, VS-SART-BL, VS-SART-EL, VS-SART-BB, and FDK. From the sinogram of the Shepp-Logan phantom, 180 projections from 360° degree beam angle was sampled for reconstruction. It is seen from the figure that the image quality of all the algorithms are improved as the number of iterations increased. For a given number of iterations, however, VS-SART-BL always outperformed the conventional SART as well as VS-SART-EL and VS-SART-BB always outperformed VS-SART-BL and the conventional SART. There was no visual difference in the image quality of VS-SART-EL and VS-SART-BB. The features of FDK were generally dimmer than SART type algorithms especially at high number of iterations.

**Figure 2:**
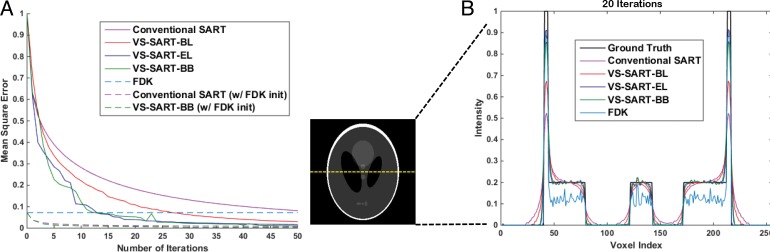
**(A)** MSE of the four algorithms as a function of number of iterations and **(B)** Line profiles of the midline of the Shepp-Logan phantom image with the four SART algorithms. For conventional SART α = 1.2. Results of FDK is also presented for comparison. 180 projections were used with 20 iterations.

Figure 2(A) shows the mean-square error (MSE) of the four SART algorithms with increase in number of iterations. All SART algorithms showed a decrease in MSE as they iterate more loops. As seen in Figure 1, VS-SART-BL showed a faster decrease in MSE than the conventional SART. Likewise, VS-SART-EL and VS-SART-BB showed even faster decrease in MSE than VS-SART-BL. VS-SART-EL and VS-SART-BB presented spiky MSE curve since they choose step sizes that are highly adaptive at each iteration. VS-SART-BB did not monotonically decrease the MSE, however, provided the best performance especially with lower number of iterations. Figure 2(B) shows line profiles of the Shepp-Logan phantom with 20 iterations. In line with the above-mentioned characteristics, VS-SART-BL performed better than the conventional SART, as well as VS-SART-EL and VS-SART-BB performed better than VS-SART-BL. At 20 iterations, VS-SART-EL and VS-SART-BB were able to follow along the features of the ground truth image with very minimal error. Only VS-SART-EL and VS-SART-BB had lower MSE than FDK, however, features presented in line profiles showed that the contrast ratio of FDK was very poor.

Table 1 demonstrates the computational complexities of the four SART algorithms. The number of forward and backward projections can be derived from the formulations presented in the method section of this paper. In terms of computational complexity per iteration, VS-SART-BL and VS-SART-EL were similar and the conventional SART and VS-SART-BB were similar, which were reflected to the actual processing time per iteration. Figure 1 showed that 20 iterations of VS-SART-EL and VS-SART-BB provided close to converged images, which is approximately 2 minutes for VS-SART-BB. Note that these numbers only represent the efficiency of each algorithm per iteration, but does not take into account the efficiency of each algorithm as a whole (e.g., does not consider the rate of convergence per iteration). To compare algorithms in a fair manner, we should limit the number of iterations of each algorithm for a given amount of time.

**Table 1:** Computational complexities of the four SART type algorithms. 180 projections from the Shepp-Logan phantom

Algorithm	# forward projection(s)	# backward projection(s)	Step size computation	Time/Iteration (Sec)
Conventional SART	1	1	None	6.047
VS-SART-BL	2	1	Armijo Line Search	10.187
VS-SART-EL	2	1	Vector Operation	10.083
VS-SART-BB	1	1	Vector Operation	7.032

**Figure 3:**
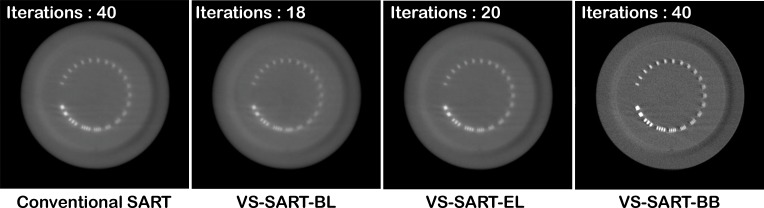
Reconstructed CatPhan 600 phantom images using the four SART algorithms The number of iterations for each algorithm was set to not exceed 230 seconds.

**Figure 4:**
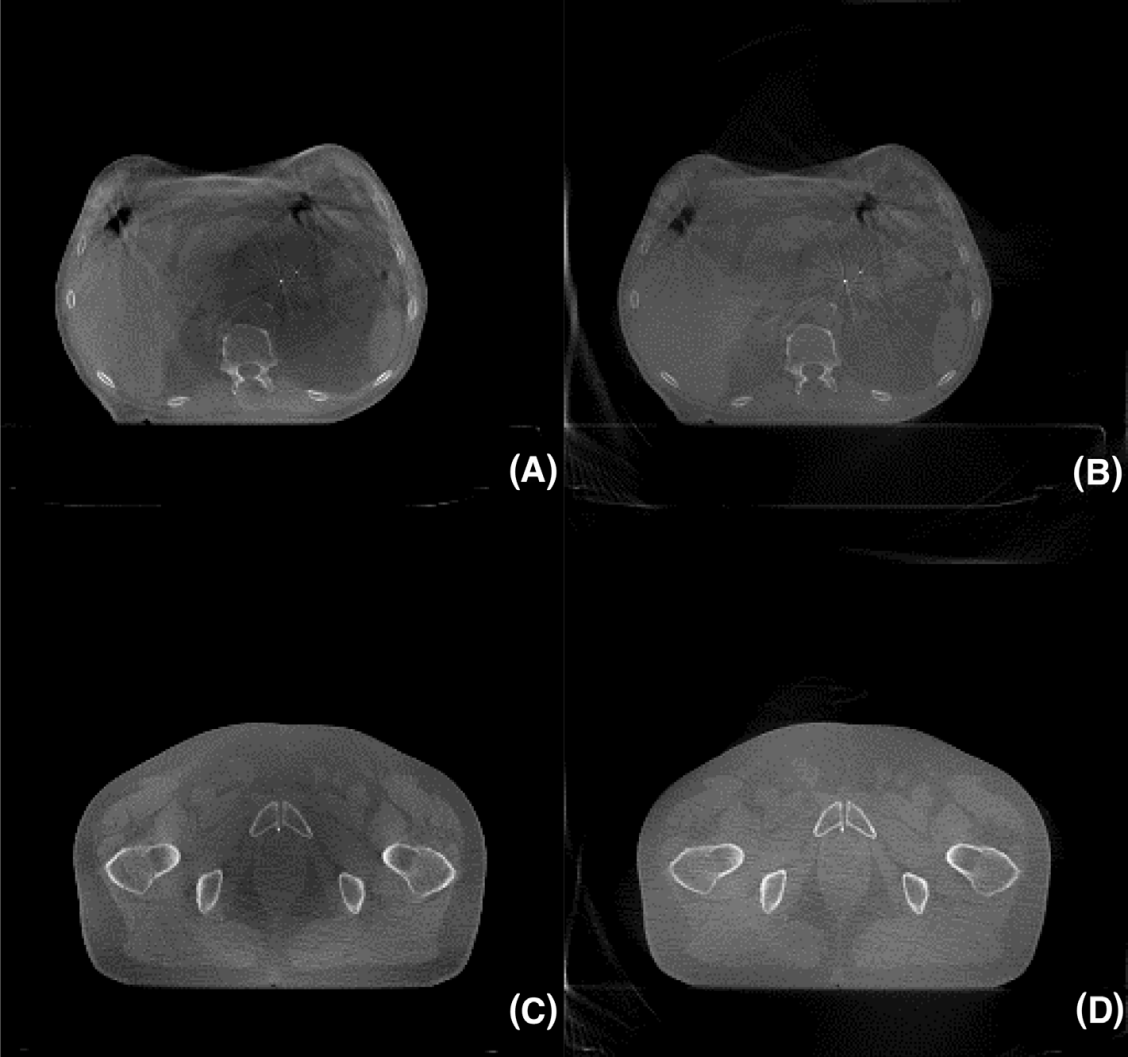
Reconstruction images of pancreatic **(A), (B)** and prostate **(C), (D)** cancer patient treated under radiation therapy. **(A)**, **(C)** FDK, **(B)**, **(D)** VS-SART-BB. A total of 655 x-ray projections were acquired in half-fan mode.

Figure 3 shows the reconstructed CatPhan 600 images of the four SART algorithms in a given time of approximately 230 seconds. Since VS-SART-BL and VS-SART-EL were slower than the conventional SART and VS-SART-BB, they were only able to run approximately 20 iterations while the other two algorithms run 40 iterations. Note the quality of the conventional SART is not any greater than VS-SART-BL or VS-SART-EL although it iterated roughly twice more than the two algorithms. VS-SART-BB clearly provided the best image quality in the given timeframe. As can be also seen from Figure 4, the image quality of VS-SART-BB outperforms the FDK algorithm in terms of artifacts that is shown at FDK as the resultant of beam hardening. Although VS-SART-BB has similar convergence rate compared to VS-SART-EL, its computational complexity is superior than VS-SART-EL, and hence it is why we see such a result. One drawback of the BB algorithm is its non-monotonic nature in reducing the cost function as seen in Figure 2(A). However, with a sufficiently high number of iterations to ensure convergence this is of a minor issue.

## DISCUSSION

We can rank order the performance of the four SART type algorithms compared in this study to be: VS-SART-BB>VS-SART-EL>VS-SART-BL>Conventional SART. With the conventional SART, the major pitfall of using a constant step size $\alpha^{\left(i\right)}$ is that it is not convergence proofed and often requires too many iterations to acquire a good image quality. VS-SART-BL ensures convergence with faster rate than the conventional SART as it employs an adaptive step size $\alpha^{\left(i\right)}$, however, still converges relatively slowly than VS-SART-EL and VS-SART-BB. This is because testing for Armijo inequality does not equate testing whether the step size is optimum. The Armijo inequality only oversees the step size to be within a reasonable range so that the solution can converge. By contrast, VS-SART-EL and VS-SART-BB look for an optimum step size and thus are much faster than VS-SART-BL. VS-SART-EL, as seen with its formulation, uses the first derivative of $f(x^{\left(i\right)}+\alpha p^{\left(i\right)})$ respect to $\alpha^{\left(i\right)}$ to find an optimum $\alpha^{\left(i\right)}$ by leveraging the quadratic characteristic. This step requires two projections to be performed per iteration. VS-SART-BB, however, requires only one projection per iteration, rendering the processing time to be twice faster than VS-SART-EL. This is a significant reduction in time, or a significant increase in number of iterations if times were set equal.

It is worth mentioning that the modern iterative reconstruction methods utilizing sparsifying transforms (e.g., L1 norm or TV) have the advantage of reducing the imaging dose. With the introduction of a regularization parameter, those algorithms are specifically suited when only few number of x-ray projections are available. However, the regularization operator that suppresses noise is also applied to anatomical features that need to be preserved. Thus, there is always a tradeoff between noise suppression and resolution preservation. Studies have indicated that a sufficient number of projections are required to reconstruct images with minimal streak artifacts for subtle anatomy [16]. This means that SART, which does not use the regularization term, is better a choice for higher number of projections considering the computational burden of regularization.

Overall, SART type algorithms benefit from an iterative process, in the sense that noise (i.e., mainly streak artifacts) is significantly reduced compared to the FDK algorithm. This enables SART algorithms to utilize fewer number of projections than FDK while still acquiring a reasonably good image quality for real-time applications. Even though we have set $x^{\left(0\right)}=0$ in our study for easier comparison, $x^{\left(0\right)}$ can be initialized with FDK and even fewer number of iterations would be needed to produce a well reconstructed image. As presented in Figure 2, FDK initialized SART algorithms converge rapidly within approximately 10 iterations.

There are several challenges associated with practical implementation of SART algorithms, one of them being imaging organs with motion. 4D-CBCT reconstruction algorithm would need to factor in time domain into its cost function. Rather than independently reconstructing 3D-CBCT in series, use of correlative information would benefit the reconstruction process. However, complexity would be the main obstacle and a modified algorithm that is computationally efficient and robust to motion artifacts will need to be devised.

## CONCLUSION

We have evaluated the image quality and computational complexity of several SART type algorithms for CBCT reconstruction. Using the Shepp-Logan phantom and CatPhan 600 phantom, we identified the slow convergence nature of the conventional SART algorithm. VS-SART-BB which adopts the efficient Barzilai-Borwein method for calculating the step size showed its superior performance over the conventional SART, VS-SART-BL, and VS-SART-EL. Using a GPU, we obtained high quality reconstructed images of Shepp-Logan phantom using 180 CBCT projections with less than 20 iterations within 2 minutes. Its enhanced computational cost allows for more iterations to be performed in a given time. We anticipate that our GPU friendly version of VS-SART-BB algorithm has the potential to handle CBCT reconstructions in a clinically feasible timeframe.

## MATERIALS AND METHODS

### Conventional SART

An iterative image reconstruction technique takes either an algebraic approach or a statistical approach. Algebraic CBCT reconstruction algorithms formulate the following algebraic equations using the X-ray projection data and solve them using iterative techniques:

Ax−b=0 (1)

where $x\in R^{N}$ denotes the unknown CBCT volume image, $A\in R^{M\times N}$ denotes the forward projection matrix (i.e., Radon transform operator), and $b\in R^{M}$ is the measured projections data. The well-known SART method solves (1) by conducting the iterations given by [8, 17]

$$\begin{array}{cc} x_{n}^{\left(i+1\right)}=x_{n}^{\left(i\right)}-\alpha^{\left(i\right)}\frac{1}{a_{+n}}\sum_{m=1}^{M}\frac{a_{m,n}}{a_{m+}}\left(a_{m}x^{\left(i\right)}-b_{m}\right) & \text{for all }1\leq n\leq N \end{array}$$ (2)

where $x_{n}^{\left(i\right)}$ denotes the *n*-th element x at iteration *i*, $0<\alpha^{\left(i\right)}<2$ is a relaxation parameter, $a_{m,n}$ is the (*m, n*)-th element of **A**, $a_{+n}=\sum_{m=1}^{M}a_{m,n}$ and $a_{m+}=\sum_{n=1}^{N}a_{m,n}$.

On the other hand, the statistical method takes a statistical estimation that considers the noisy nature of X-ray projections. This results in the following weighted least squares problem

$$\underset{x\geq 0}{\min}\;{\left\|Ax-b\right\|}_{W}^{2}=\underset{x\geq 0}{\min}\;{\left(Ax-b\right)}^{T}W(Ax-b).$$ (3)

whose weight matrix **W** can be determined by various weighting strategies proposed in the past [18, 19]. This equation can be solved with various types of non-linear optimization algorithms.

We show that the SART algorithm (2) is essentially an iterative algorithm for solving the following weighted least squares problem [10]:

$$\underset{x\geq 0}{\min}f\left(x\right)=\underset{x\geq 0}{\min}\;{\left(Ax-b\right)}^{T}W_{r}^{-1}(Ax-b)$$ (4)

where $W_{r}$ is an M×M diagonal matrix whose *m*-th diagonal element is the *m*-th row sum ($a_{m+}$) of the forward projection matrix **A**. To see this, we first stack the equations in (2) together and succinctly express as

$$x^{\left(i+1\right)}=x^{\left(i\right)}-\alpha^{\left(i\right)}W_{c}^{-1}A^{T}W_{r}^{-1}(Ax^{\left(i\right)}-b)$$ (5)

where $W_{c}$ is an N×N diagonal matrix whose *n*-th diagonal element is the *n*-th row sum ($a_{n+}$) of the backward projection matrix $A^{T}$. If we ignore $W_{c}^{-1}$ for now, it can be easily seen that (5) is essentially a gradient descent algorithm for solving (4) as the gradient of f(x) is computed as

$$\nabla f(x)=A^{T}W_{r}^{-1}(Ax-b).$$ (6)

This further implies that eq. (5) is simply a variant of a gradient descent algorithm that employs

$$s^{\left(i\right)}\equiv W_{c}^{-1}\nabla f(x^{\left(i\right)})$$ (7)

to a descent direction^1^ to solve (4), demonstrating the equivalence of the algebraic SART-type approach and the statistical weighted least squares approach.

This connection motivates us to interpret the SART relaxation parameter $\left\{\alpha^{\left(i\right)}\right\}$ as the step-size of a gradient descent iteration. The conventional SART method that chooses a constant $\alpha^{\left(i\right)}=\alpha \;(0<\alpha <2)\forall \;i$ can be considered as a constant step size gradient descent algorithm.

### VS-SART-BL

We now present the variable step size SART-type (VS-SART) algorithms that choose different $\alpha^{\left(i\right)}$'s to accelerate the convergence of the SART-type algorithms. For the rest of the paper, we consider the projected SART descent direction $p^{\left(i\right)}$ given by

$$\begin{array}{ll} p_{n}^{\left(i\right)}=s_{n}^{\left(i\right)} & \text{if }s_{n}^{\left(i\right)}\leq 0\text{ or }x_{n}^{\left(i\right)}\geq 0\\ p_{n}^{\left(i\right)}=0 & \text{otherwise}. \end{array}$$ (8)

to effectively incorporate the non-negativity constraint in (4).

We begin with considering two conventional approaches for selecting $\alpha^{\left(i\right)}$. The first approach is a backtracking line search method (VS-SART-BL). Let $p^{\left(i\right)}$ denote the search direction at iteration *i*. The algorithm finds the largest $\alpha \in \left\{\alpha_{\max},\beta \alpha_{\max},\beta^{2}\alpha_{\max},\ldots \right\}$ that satisfies the Armijo condition given by

$$f(x^{\left(i\right)}-\alpha p^{\left(i\right)})\leq f(x^{\left(i\right)})-\sigma \alpha \nabla f{\left(x^{\left(i\right)}\right)}^{T}p^{\left(i\right)}$$ (9)

where β∈(0,1) and $\sigma \in (0,\frac{1}{2})$. Once the proper $\alpha^{\left(i\right)}$ is found, eq. (5) becomes

$$x^{\left(i+1\right)}={\left[x^{\left(i\right)}-\alpha_{BL}^{\left(i\right)}p^{\left(i\right)}\right]}^{+}$$ (10)

where ${\left[x\right]}^{+}\equiv \max(x,0)$ is used to incorporate the non-negativity constraint in problem (4). Regarding the complexity, it can be easily seen that just one projection operation is sufficient to find $\alpha^{\left(i\right)}$. As inequality (9) is equivalent to

$$\alpha^{2}||W_{r}^{-\frac{1}{2}}Ap^{\left(i\right)}|{|}_{2}^{2}-2\alpha {\left(p^{\left(i\right)}\right)}^{T}\nabla f(x^{\left(i\right)})+\sigma \alpha \nabla f{\left(x^{\left(i\right)}\right)}^{T}p^{\left(i\right)}\leq 0,$$ (11)

the initial check requires the computationally expensive projection once to compute $Ap^{\left(i\right)}$ to compute the first term. By storing the matrix and vector multiplication results, one can complete the subsequent checks with only scalar multiplications, greatly simplifying the backtracking line search.

### VS-SART-EL

The second approach, an exact line search (VS-SART-EL), leverages the quadratic nature of f(x) and computes the step size that minimizes $f(x^{\left(i\right)}-\alpha p^{\left(i\right)})$ given by [20]

$$\alpha^{\left(i\right)}=\frac{{\left(p^{\left(i\right)}\right)}^{T}\nabla f(x^{\left(i\right)})}{||W_{r}^{-\frac{1}{2}}Ap^{\left(i\right)}|{|}_{2}^{2}}$$ (12)

and the VS-SART-EL iteration becomes

$$x^{\left(i+1\right)}={\left[x^{\left(i\right)}-\alpha_{EL}^{\left(i\right)}d^{\left(i\right)}\right]}^{+}$$ (13)

where $d^{\left(i\right)}\equiv W_{c}p^{\left(i\right)}$. Note $d^{\left(i\right)}$ is used instead of $p^{\left(i\right)}$ as (12) is valid in the absence of $W_{c}^{-1}$. Similar to VS-SART-BL, VS-SART-EL requires one projection operation to compute the denominator in (12), indicating that their computational complexity is in the same order.

### VS-SART-BB

**Figure 5:**
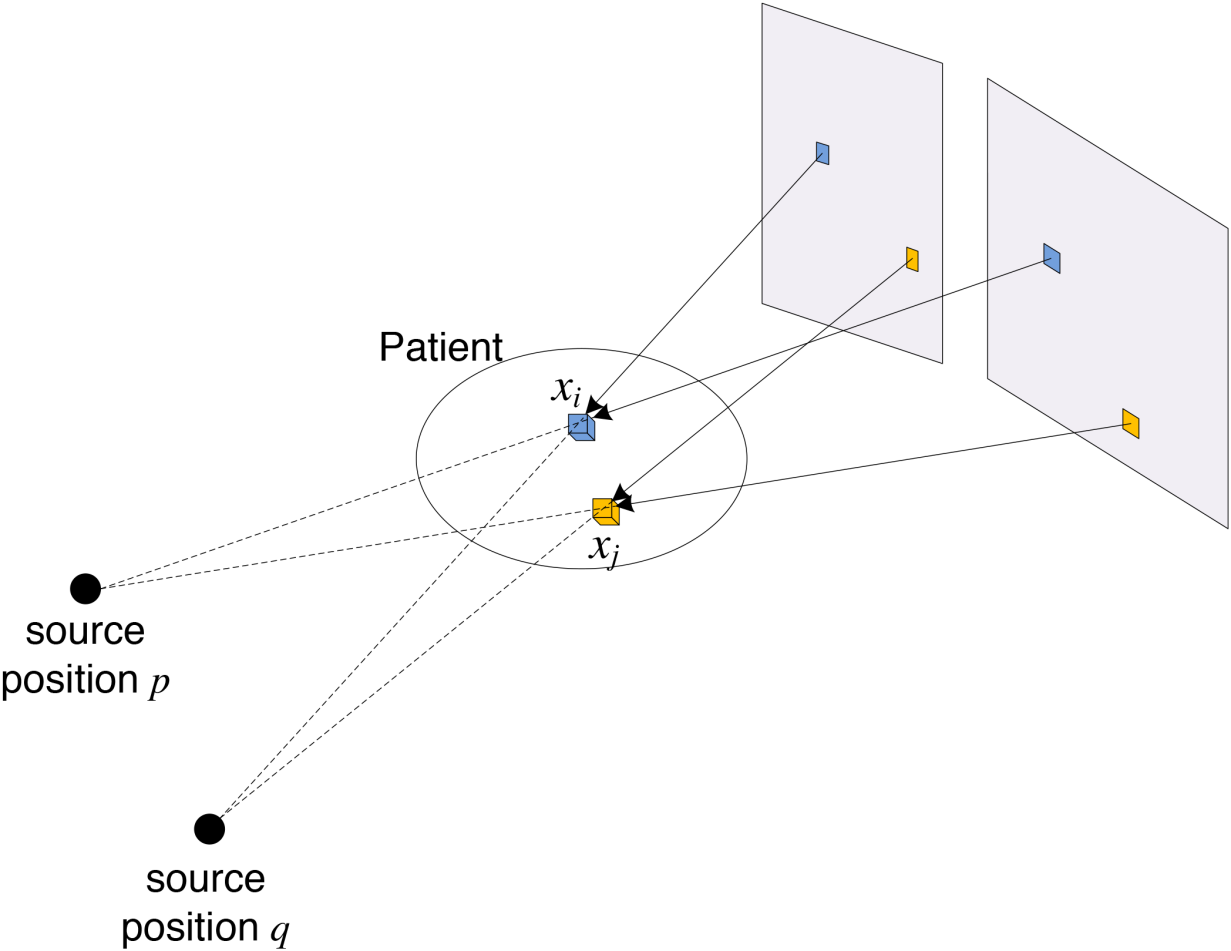
Reconstructed images as a function of number-of-projections and number-of-iterations The window and level were kept the same for all images.

In this paper, we further investigate the geometric structure of the problems (3) and (4) and propose a step-size determination method for exploiting that structure. The *n*-th column of matrix **A** represents the backprojection operation from different detector pixels to the *n*-th voxel. As illustrated in Figure 5, two different voxels ($x_{i}$ and $x_{j}$) are backprojected from different sets of detector pixels. Therefore, $a_{i}$ and $a_{j}$ have disjoint sets of non-zero positions, resulting in $a_{i}{}^{T}a_{j}=0$. This orthogonal relationship holds for predominant cases (i.e., unless two voxels are adjacent to each other and backprojected by one or more same detector pixels), and suggests that $A^{T}$ and **A** are quasi-orthogonal. Therefore, the Hessian matrices in (3) and (4), $A^{T}A$ and $A^{T}W_{r}^{-1}A$, can also be approximated by a diagonal matrix. Based on this observation, we propose to use the Barzilai-Borwein method (VS-SART-BB) to determine the step size [14, 15, 21, 22]. Let $I_{N}$ denote the N×N identity matrix. The Barzilai-Borwein method approximates the Hessian at iteration *i* by $H=\eta^{\left(i\right)}I_{N}$ and finds the scalar $\eta^{\left(i\right)}$to approximate the true Hessian by approximately solving the Secant condition in the quasi-Newton method as

$$p^{\left(i\right)}-p^{\left(i-1\right)}\approx \eta^{\left(i\right)}(x^{\left(i\right)}-x^{\left(i-1\right)})$$ (14)

(or $d^{\left(i\right)}-d^{\left(i-1\right)}\approx \eta^{\left(i\right)}(x^{\left(i\right)}-x^{\left(i-1\right)})$)

The least squares solution is given by

$$\eta^{\left(i\right)}=\frac{{\left(x^{\left(i\right)}-x^{\left(i-1\right)}\right)}^{T}(p^{\left(i\right)}-p^{\left(i-1\right)})}{{\left\|x^{\left(i\right)}-x^{\left(i-1\right)}\right\|}_{2}^{2}}.$$ (15)

(or $\eta^{\left(i\right)}=\frac{{\left(x^{\left(i\right)}-x^{\left(i-1\right)}\right)}^{T}(d^{\left(i\right)}-d^{\left(i-1\right)})}{{\left\|x^{\left(i\right)}-x^{\left(i-1\right)}\right\|}_{2}^{2}}$)

Then, once $\eta^{\left(i\right)}$ is calculated, the VS-SART-BB iteration is given by

$$x^{\left(i+1\right)}={\left[x^{\left(i\right)}-{\left(\eta^{\left(i\right)}\right)}^{-1}p^{\left(i\right)}\right]}^{+}.$$ (16)

(or, equivalently, $x^{\left(i+1\right)}={\left[x^{\left(i\right)}-{\left(\eta^{\left(i\right)}\right)}^{-1}d^{\left(i\right)}\right]}^{+}$)

As $\eta^{\left(1\right)}$ is arbitrary, we set $\eta^{\left(1\right)}=\alpha_{EL}^{\left(1\right)}$ in this paper. Note that some surprising super-linear convergence results are reported in [14] and its convergence for quadratic functions is proved in [21, 22].

The theoretical advantage of VS-SART-BB over VS-SART-BL and VS-SART-EL is that eq. (15) does not require any computationally expensive projection operation, indicating less computations in each operation. Moreover, for a given number of iterations, the aforementioned geometric structure of problems (3) and (4) motivates us to investigate whether even faster convergence can be achieved with VS-SART-BB.

In order to effectively handle the large dimension of the iterative CBCT reconstruction problem, all algorithms are implemented using a parallel processing hardware and the associated reconstruction time is recorded. In addition to Intel Core^TM^ i7 CPU with 2.68 GHz clock speed, and 12.0 GB DDR3 RAM on a 64-bit Vista OS, we used a single GTX 295 card (NVIDIA, Santa Clara, CA) that consists of 480 processing cores with 1.24 GHz clock speed and 1,792 MB memory. The most computationally intensive forward and backward projection operations are implemented using the GPU in CUDA C/C++ programming (NVIDIA, Santa Clara, CA). Computational tasks involving Radon transform were parallelized including the forward projection **A**, backward projection $A^{T}$, and vector operations such as $p^{\left(i\right)}$ and Ax−b. Each detector pixel of **A** was assigned to one GPU thread. Then, the image voxels along the path between the source and the detector were summed independently for each detector pixel. For $A^{T}$, each image voxels were assigned to a GPU thread. Vector operations were implemented in a similar fashion.

We applied SART algorithms to Shepp-Logan numerical phantom, CatPhan 600 physical phantom (The Phantom Laboratory, Salem, NY), and pancreatic and prostate cancer patient using TrueBeam^TM^ system (Varian Medical Systems, Palo Alto, CA) from a 360° beam angle. The imager has 1024×768 detector pixels of size 0.388×0.388 mm^2^. In this paper, we downsampled this to 512×384 pixels of size 0.776×0.776 mm^2^ for reconstruction. The reconstruction volume of 512×512×70 voxels, each of dimension 0.49×0.49×0.2 mm^3^, is vectorized to construct **X**.

## Footnotes

1 Weighted gradient (by positive weights) produces a descent direction.
